# Is the supine position associated with loss of airway patency in unconscious trauma patients? A systematic review and meta-analysis

**DOI:** 10.1186/s13049-015-0116-0

**Published:** 2015-07-01

**Authors:** Per Kristian Hyldmo, Gunn E Vist, Anders Christian Feyling, Leif Rognås, Vidar Magnusson, Mårten Sandberg, Eldar Søreide

**Affiliations:** Department of Research and Development, Norwegian Air Ambulance Foundation, Drøbak, Norway; Department of Anesthesiology and Intensive Care Medicine, Sørlandet Hospital, Kristiansand, Norway; The Norwegian Knowledge Center for the Health Services, Oslo, Norway; Department of Anesthesiology, Oslo University Hospital, Oslo, Norway; Pre-hospital Critical Care Services, Aarhus, Denmark; Department of Anesthesiology, Landspitalinn University Hospital, Reykjavík, Iceland; Faculty of Medicine, University of Oslo, Oslo, Norway; Air Ambulance Department, Oslo University Hospital, Oslo, Norway; Network for Medical Sciences, University of Stavanger, Stavanger, Norway; Department of Anesthesiology and Intensive Care Medicine, University Hospital of Stavanger, Stavanger, Norway

**Keywords:** Airway management, Airway obstruction, Airway patency, Trauma care, Complications, Supine position, Recovery position, Patient safety

## Abstract

**Background:**

Airway compromise is a leading cause of death in unconscious trauma patients. Although endotracheal intubation is regarded as the gold standard treatment, most prehospital providers are not trained to perform ETI in such patients. Therefore, various lateral positions are advocated for unconscious patients, but their use remains controversial in trauma patients. We conducted a systematic review to investigate whether the supine position is associated with loss of airway patency compared to the lateral position.

**Methods:**

The review protocol was published in the PROSPERO database (Reg. no. CRD42012001190). We performed literature searches in PubMed, Medline, EMBASE, Cochrane Library, CINAHL and British Nursing Index and included studies related to airway patency, reduced level of consciousness and patient position. We conducted meta-analyses, where appropriate. We graded the quality of evidence with the GRADE methodology. The search was updated in June 2014.

**Results:**

We identified 1,306 publications, 39 of which were included for further analysis. Sixteen of these publications were included in meta-analysis. We did not identify any studies reporting direct outcome measures (mortality or morbidity) related to airway compromise caused by the patient position (lateral vs. supine position) in trauma patients or in any other patient group. In studies reporting only indirect outcome measures, we found moderate evidence of reduced airway patency in the supine vs. the lateral position, which was measured by the apnea/hypopnea index (AHI). For other indirect outcomes, we only found low or very low quality evidence.

**Conclusions:**

Although concerns other than airway patency may influence how a trauma patient is positioned, our systematic review provides evidence supporting the long held recommendation that unconscious trauma patients should be placed in a lateral position.

**Electronic supplementary material:**

The online version of this article (doi:10.1186/s13049-015-0116-0) contains supplementary material, which is available to authorized users.

## Background

According to the World Health Organization, airway compromise is a leading cause of death during the first hours after trauma [1]. Thus, early endotracheal intubation (ETI) has been recommended for unconscious trauma patients [2-4]. However, on a global scale, most prehospital providers are not trained to perform ETI. Furthermore, prehospital ETI has been questioned because of the potential complications [5-8].

**Figure 1:**
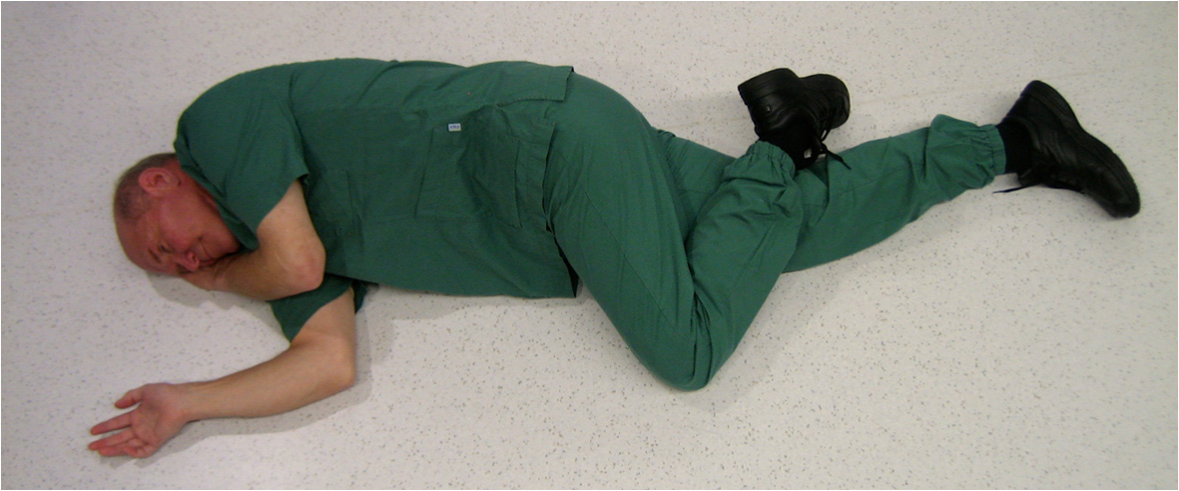
The recovery position.

**Figure 2:**
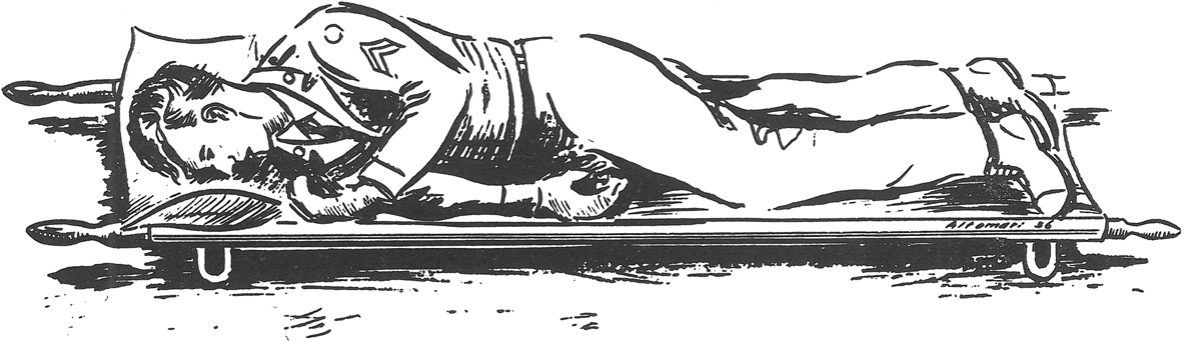
The NATO coma position.

**Figure 3:**
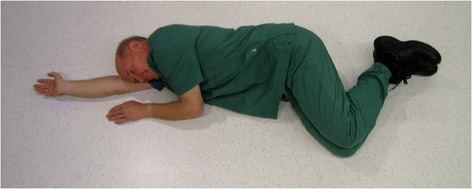
The HAINES position.

**Figure 4:**
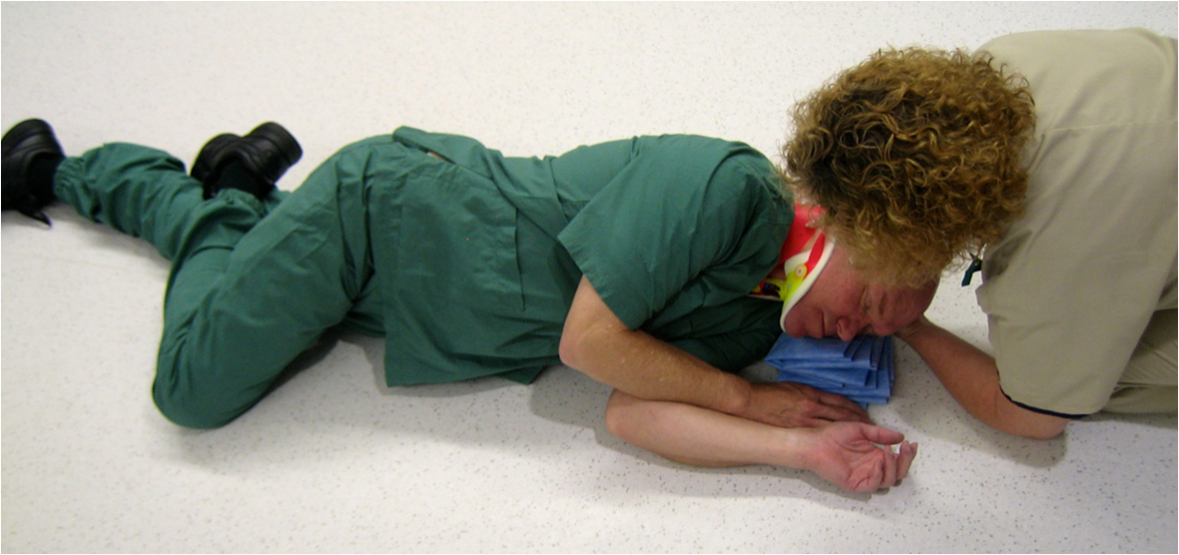
The lateral trauma position.

For decades, placing an unconscious, non-intubated patient in the lateral position (“recovery position”, Figure 1) has been recommended to maintain an open airway, which is also true for trauma [9-12] (Figure 2). However, due to the fear of worsening a potential cervical spine injury, clinical guidelines and authoritative training manuals dictate that such patients should be transported in the supine position while strapped to a spine board, with a cervical collar in place [13]. Attempting to balance these two considerations, various authors have proposed the use of adapted lateral positions [14-17] (Figures 3 and 4). The lateral trauma position (LTP) has, to some extent, been implemented in clinical practice [17]. However, the positioning of unconscious trauma patients is still a controversial issue worldwide, with both medical and medico-legal implications.

We conducted a systematic review to answer the following question: In the unconscious trauma patient, is the supine position associated with a loss of airway patency compared to the lateral position?

## Methods

The protocol for this review was published in the PROSPERO database for systematic reviews [18]. We used the PICO (Population, Intervention, Comparison and Outcome measures) format to develop our research question and search strategies [19]. Furthermore, we used the PRISMA checklist (Preferred Reporting Items for Systematic Reviews and Meta-Analyses) [20] as a guide to ascertain the quality of the review process and manuscript. Written informed consent was obtained from the models for publication of the accompanying images.

### Inclusion criteria

#### Types of participants

Our main question focused on unconscious trauma patients; however, due to the expected paucity of studies specifically dealing with unconscious trauma patients, we decided to include all patients with a reduced level of consciousness (LOC), regardless of the cause and patient location.

#### Types of interventions and comparisons

We defined the lateral position as the intervention, which was compared with the supine position. There are many forms of lateral position that are used in the medical literature. However, we did not restrict the intervention to any specific lateral position or to how the patient was placed in that position.

Because the study question was specifically linked to the supine position, we also included studies addressing the effect of a reduced LOC on airway patency in the supine position alone.

#### Outcome measures

We aimed to use patient mortality (short and long term) and morbidity (e.g., aspiration, aspiration pneumonia or the Glasgow outcome scale) as the measured outcomes in our analysis. However, due to the lack of studies reporting these variables, we included the following indirect airway patency outcome measures: hypoxia, hypercapnia, hypoventilation, stridor score, apnea/hypopnea index (AHI), respiratory disturbance index (RDI), upper airway resistance (Rua) and work of breathing (WOB). If a study reported multiple relevant outcome measures, we included all of them.

#### Study types

We included all study designs that used a control or comparison group, including crossover studies where patients/volunteers acted as their own controls.

#### Search methods used to identify the studies

We searched the following databases: PubMed, Medline, EMBASE, Cochrane Library, CINAHL and British Nursing Index. We modified the terms when searching different databases, as necessary. We also performed forward and backward citation searches and manual searches of “gray” literature, such as relevant textbooks. No limits on the publication date or language were applied. The searches were updated in June 2014. Combinations of the following words and their variations were sought:

- unconscious, Glasgow Coma Scale, coma, craniocerebral trauma, brain injury, sleep apnea
- patient positioning, supine position, spine-/backboard, vacuum mattress
- airway obstruction, anoxia, hypoxia, hypoventilation, hypercapnia, mortality, morbidity, Glasgow Outcome Scale

The full search strategies for all the databases searched are described in Additional file 1.

### Data collection and analysis

The principal investigator (PKH) assessed all studies that were identified in the searches. The remaining authors each assessed one portion of the studies, thus two investigators independently assessed each reference. All disagreements were resolved through discussion or by consulting with a third author.

### Data extraction and management

We designed a data extraction form, with which two review authors independently extracted the data from the eligible studies. Discrepancies were resolved through discussion. We extracted the following data: the first author, publication year, population, intervention and comparison details, measured outcome, measurement time and measurement method. For studies relevant to the meta-analysis, the first author entered the outcome data into the Review Manager software program [21], and another author checked the data for accuracy.

### Assessing the risk of bias in the included studies

Two review authors independently assessed the risk of bias for each study using the criteria outlined in the Cochrane Handbook for Systematic Reviews of Interventions [22] or the checklists from the Norwegian Knowledge Centre for the Health Services [23]. We resolved any disagreement by discussion or by including a third assessor.

The risk of bias was assessed according to the sequence generation, allocation concealment, selection of groups and group comparability, blinding of the participants, provider and assessor, and incomplete outcome data, including possible attrition bias and selective reporting bias.

### Measuring the treatment effect

#### Dichotomous data

We planned to present the results as a summary risk ratio (RR) with 95% confidence intervals (CI) [19]. However, no such data were identified.

#### Continuous data

We reported the mean difference (with standard deviations) when the outcomes were measured in the same manner between the trials.

### Analysis

Where appropriate, we combined the results from the different studies included in a meta-analysis. We performed the statistical analysis using RevMan [21] software. Expecting differences between trials, we used random-effects meta-analysis as the default method to combine the data. We used the generic inverse-variance method available in RevMan to perform the analysis. In cases in which it was inappropriate to combine the results, we descriptively present the results in tables.

### Missing data

For the included studies, we noted the level of attrition, if any. Most of the included studies were crossover studies that used the patients as their own controls; in most of these studies, there was no attrition. In the remaining studies attrition was negligible, and we performed no further analyses. For the continuous measures, we used actual measurements (no imputations).

### Assessment of the heterogeneity

We examined the meta-analysis forest plot for heterogeneity among studies, and considered the size and direction of the effect, using I^2^ statistics to quantify the level of heterogeneity. We recommend exercising caution in interpreting the results when unexplained heterogeneity is substantial or considerable (i.e., I^2^ between 30 and 60% or between 50 and 100%, respectively).

### Assessing the studies that were not applicable to the meta-analysis

Most of the outcomes in these studies were insufficiently reported, so that they cannot be included in analysis. We present these studies according to the measured outcomes, which are described in the tables. Differences are noted as favoring one of the positions, designated by “+” for favoring the intervention (the lateral position) or “-“ for favoring the control (the supine position), and by “?” when the direction is unclear or not significant in favor of one of the positions.

### Grading the quality of evidence

We used the GRADE methodology to grade the quality of evidence for each of the critically important outcomes with sufficient results presented [24]. For each outcome, the quality of the evidence was assessed using the eight GRADE criteria: five criteria for downgrading (study limitations, heterogeneity, indirectness of the evidence, imprecision, and reporting bias) and three criteria for upgrading (large effect, dose–response gradient, and plausible confounding). The outcomes that were insufficiently reported are associated with large uncertainty and should be interpreted with caution.

## Results

**Figure 5:**
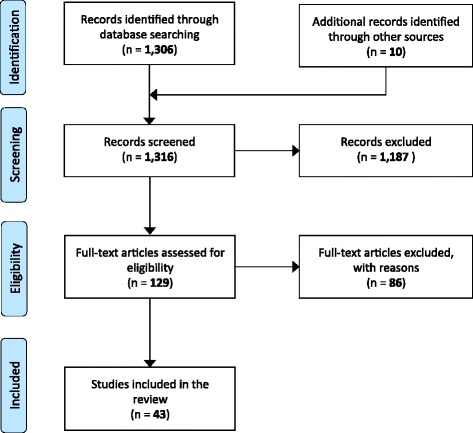
Inclusion and exclusion of studies.

We did not identify any specific studies involving unconscious trauma patients and airway patency using patient mortality or morbidity as the measured outcomes. In addition, we did not find any trauma patient studies reporting indirect outcomes. Broadening the inclusion criteria to all patients with reduced consciousness, we still did not identify any studies reporting direct outcome measures (i.e., mortality or morbidity). However, when including studies that reported indirect outcome measures, we identified 1,316 unique publications, of which 43 were included for further analysis (Figure 5). Some of the included studies were randomized control trials (RCTs) that were designed to evaluate other interventions, but they included baseline data that were useful for our comparisons. We included these baseline data comparisons as observational data in our review.

**Figure 6:**
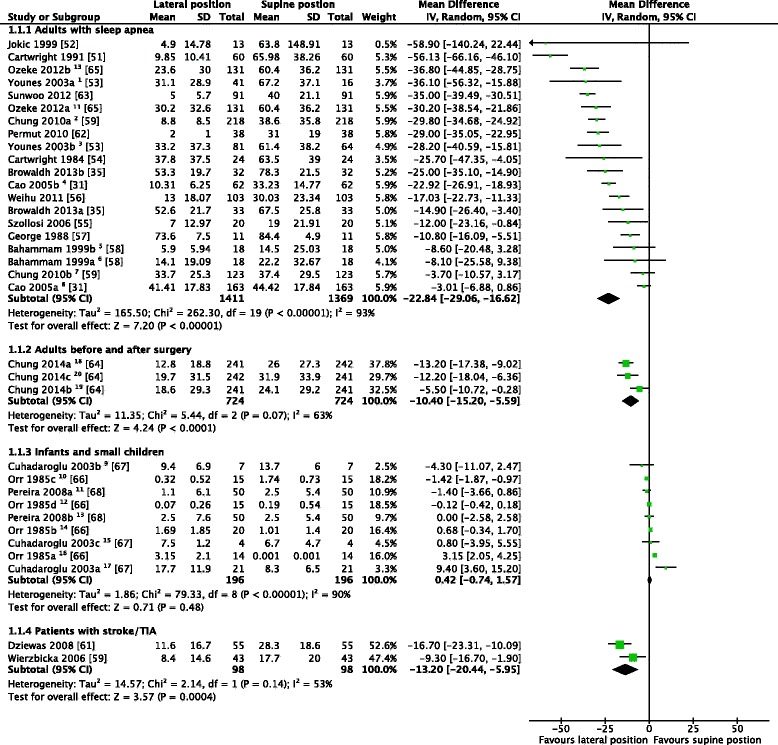
We included 20 studies with a total of 34 comparisons in the meta-analysis. Some of the studies reported data from more than one group: REM-sleep^1, 6^; non-REM sleep^3, 5^; positional obstructive sleep apnea (OSA)^2, 4, 18^; non-positional OSA^7, 8^; with tonsillo-andenomegaly^9^; with adenoid hypertrophy^17^; no obstruction^15^; at 40-44 weeks post-conseptional age^16^; at 45-49 weeks post-conseptional age^14^; at 50-54 weeks post-conseptional age^10^; at 55-59 weeks post-conseptional age^12^; left lateral vs. supine position^11^; right lateral vs. supine position^13^; preoperative night^18^, first^19^ and third^20^ postoperative night.

We were able to combine the results from 20 publications (34 comparisons) in a meta-analysis (Figure 6). None of the included studies reported dichotomous outcome measures. The excluded articles and the reasons for their exclusion are summarized in Additional file 2.

### Studies investigating only the supine position

We identified five studies [25-29] comparing the airway patency of awake patients in the supine position to the airway patency of the same patients during a reduced LOC. The causes of the reduced LOC were general anesthesia, drug-induced sedation or sleep. Using different types of indirect outcome measures, all studies indicated that reduced LOC in the supine position was associated with worsened airway patency (Table 1).

**Table 1:** **Studies reporting supine awake vs. supine with reduced consciousness**

**Study (Year) country**	**Patients**	**Study outline**	**Results**	**Notes**
**Safar** ***et al.*** [25] (1959) USA	80 adult volunteers, no lung- or airway disease	Interventional study of airway patency under general anesthesia, placing the volunteers in various supine and prone positions. For the purpose of our study: Supine, awake vs. supine, anesthetized. Outcome: open, partially obstructed and obstructed airway.	Incidence of obstruction:	No p-value given.
			• Supine, awake: 0%; anesthetized: 54% partially obstructed, 36% obstructed, 10% open airway	Loss of airway patency when going from awake to general anesthesia in the supine position.
**Kopelman** ***et al.*** [26] (1986) England	40 adult male volunteers, 20 obese, 20 normal weight	Observational study of oxygen saturation while awake and during sleep, both in supine position.	Minimum S_a_O_2_, mean (%):	p < 0.01 for both comparisons.
			• Obese group: Supine, awake: 96, asleep: 80	Shows lower oxygen saturation asleep in the supine position vs. awake, most profound in the obese group.
			• Normal weight group: Supine, awake: 97, asleep: 94	
**Ikeda** ***et al.*** [27] (2006) Japan	14 healthy adult male volunteers	Observational study on airway collapsibility under midazolam sedation in supine position vs. 30 degrees elevated upper body. Outcome is critical closing pressure of upper airway (P_crit_)	P_crit_, mean, cmH_2_O (SE):	p < 0.05.
			• Elevated upper body: −13.2 (1.3)	Critical closing pressure of upper airway may be regarded as a measure of patency of the airway; the lower supine value means increased collapsibility.
			• Supine: −8.2 (1.4)	
**Lee** ***et al.*** [28] (2009) Taiwan	48 adult patients, 28 with obstructive sleep apnea (OSA).	Observational study on work of breathing (WOB) in supine position, asleep and awake. Reports data in three OSA groups and control group.	WOB, mean, J/l:	p < 0.05 for all comparisons.
			• Control group: Supine, awake: 0.70, asleep: 1.16	An increased WOB may be an indicator of airway obstruction, but no firm conclusion should be drawn from this study.
			• Eucapnic, non-obese group: Supine, awake: 1.20, asleep: 2.07	
			• Eucapnic, obese group: Supine, awake: 1.41, asleep: 2.25	
			• Hypercapnic group: Supine, awake: 2.27, asleep: 3.13	
**Tagaito** ***et al.*** [29] (2010) Japan	9 male patients with OSA	Interventional study of upper airway closing pressure during general anesthesia and sitting vs. supine position. P_close_ is estimated on to levels of the upper airway.	Airway closing pressure, P_close_, median, cmH_2_O:	p < 0.01 for both comparisons.
			• Retropalatal airway: Sitting: −3.47, supine: 2.20	Airway closing pressure may be regarded as a measure of patency of airway; the lower values in the supine group mean increased collapsibility.
			• Retroglossal airway: Sitting: −5.31, supine: 2.67	

### Studies of the lateral vs. supine positions

The included studies reported a multitude of outcome measures, including oxygen desaturation, stridor score, upper airway resistance (R_ua_), closing pressure (P_crit_ and P_close_), minute ventilation (MV) volume, RDI, and AHI. Most of these studies could not be included in the meta-analysis, but the results are summarized in terms of the direction of the effect (Tables 2, 3, 4, and 5).

**Table 2:** **Studies reporting oxygen desaturation**

**Study (Year), country**	**Patients**	**Study outline**	**Results**	**Favors lateral**	**Notes**
***Preoperative adult patients***					
**Rosenberg-Adamsen ** ***et al.*** [30] (1997) Denmark	13 patients scheduled for gastro-intestinal surgery	Descriptive sleep study of	• Mean average SpO2 (%): Supine: 95, lateral: 95	**?**	We have used preoperative values only (postoperative values may be confounded).
		• Mean average SpO_2_ supine vs. lateral sleeping during preoperative night.	• Mean number of desaturation episodes/h: Supine: 13, lateral: 3	**+**	Reports p = 0.04.
		• Mean number of desaturations pr. hour, defined as sudden desaturation of more than 5% below the patient’s baseline value.			No difference in mean SpO2, but in number of desaturation episodes.
***Adults with obstructive sleep apnea***					
**Cao** ***et al.*** [31] (2005) China	225 adults with known obstructive sleep apnea (OSA)	Descriptive sleep study of nadir (lowest) SpO_2_ in lateral vs. supine sleeping position. Reports separately on positional patients (with a known position dependent OSA) and non-positional patients.	Nadir SpO_2_ (mean; %),	**?**	p-values not given.
			• Positional patients: Supine: 78.9, lateral: 79.5		Very low values for both groups in both positions.
			• Non-positional patients: Supine: 71.5, lateral: 75.1	**+**	
**Shao** ***et al.*** [32] (2011) China	110 elderly patients with OSA	Descriptive sleep study of oxygen saturation in supine left and right sleeping positions, reporting time intervals between desaturation episodes (the latter not defined).	Time between desaturation episodes (median; min): Supine: 2.36, left side: 11.54, right side: 12.45	**+**	Conference abstract only.
					p < 0.01 for both left and right vs. supine.
**Oksenberg** ***et al.*** [33] (2000) Israel	30 adults with OSA	Descriptive sleep study, reporting	• Mean apnea duration + (sec): Supine: 26.6, lateral: 22.8	**+**	p < 0.0001
		• apnea duration	• Mean minimum SpO_2_ (%): Supine: 82.0, lateral: 86.2	**+**	Clinically relatively small differences.
		• minimum oxygen desaturation	• Mean ∆ SpO2 (%): Supine: 12.6, lateral: 8.3	**+**	
		• difference between max. and min. oxygen desaturation			
**Sasai ** ***et al.*** [34] (2011)	30 adults with OSA	Descriptive study of average SaO_2_ in supine vs. all sleeping positions. Reports data sorted by severity of OSA (moderate and severe).	Mean average SaO_2_ (%), supine vs. all:	**?**	p < 0.01 and < 0.05, respectively, but at least in the severe OSA group the differences are not clinically important.
			• Moderate OSA: Supine: 93.9, all positions: 95.1		
			• Severe OSA: Supine: 88.0, all positions: 88.4	**?**	
**Browaldh ** ***et al.*** [35] (2013) Sweden	64 OSA patients	Two groups, one treated surgically for OSA (1), the other just observed (2). Reports data on oxygen desaturation index (ODI; events/h) before treatment.	ODI (events/h):	**+**	p-values not given. Clinically important difference, may have been even larger if supine was not included in all positions.
			• 1: Supine: 62.7, all positions: 44.6		
			• 2: Supine: 54.5, all positions: 41.1	**+**

**Table 3:** **Studies reporting other outcomes**

**Study (Year) country**	**Patients**	**Study outline**	**Results**	**Favors lateral**	**Notes**
**Penzel ** ***et al.*** [39] (2001) Germany	16 male adult patients with suspected obstructive sleep apnea (OSA)	Observational sleep study reporting upper airway closing pressure in lateral and supine position during three sleep stages.	Airway closing pressure (P_crit_, cmH_2_O)	**+**	P for all < 0.05.
			• Light sleep:		Airway closing pressure is a measure of collapsibility, lower/negative pressure means less collapsibility.
			Lateral: −2.2, supine: 0.6		
			• Slow-wave sleep:		
			Lateral: −1.7, supine: 0.3		
			• REM-sleep:		
			Lateral: −2.2, supine: 1.2		
**Isono** ***et al.*** [40] (2002) Japan	8 male patients with OSA under evaluation for surgery	Observational study with patients anesthetized and airway closing pressure measured in lateral and supine positions at two areas (retropalatal and retroglossal airway). Airway pressure (P_AW_, cmH_2_O) was measured to cessation of air passage. This P_AW_ equals the airway closing pressure, P_crit_.	Airway closing pressure (P_crit_, cmH_2_O)	**+**	For both areas: p < 0.05.
			• Retropalatal airway:		Airway closing pressure is a measure of collapsibility, lower/negative pressure means less collapsibility.
			Lateral: −1.86, supine: 2.05		
			• Retroglossal		
			• airway:		
			Lateral: −3.17, supine: 0.49		
**Jordan ** ***et al.*** [38] (2003) Australia	33 healthy, nonsmoking adult volunteers	Polysomnographic study (PSG) study reporting baseline inspiratory minute ventilation (MV) and upper airway resistance (R_ua_) in left lateral and supine position.	• MV_insp_, mean (l/min):	**?** **+**	MV: Small differences, may not be clinically important.
			Men: Lateral: 7.5, supine: 7.0		
			Women: Lateral: 5.9, supine: 6.0		
			• R_ua_, mean (cmH_2_O/l)		R_ua_: Higher airway resistance in supine position. Reports “significantly difference”, no p-value.
			Men: Lateral: 4.1, supine: 5.8		
			Women: Lateral: 3.4, supine: 6.6		
**Arai ** ***et al.*** [36] (2004) Japan	30 children (1–10 years) with OSA, scheduled for ear-nose-throat (ENT) surgery.	Observational study of airway obstruction in general anesthesia, in lateral and supine position, using stridor score (1: normal, 4: no airway sound detected)	Stridor score, median:	**+**	p < 0.05
			Lateral: 3, supine: 4		Crude but clinically important outcome.
**Litman** ***et al.*** [41] (2005) USA	17 children (2–11 years), scheduled for MRI.	Observational study of total upper airway volume in left lateral and supine position, using MRI.	V_upper airway_, mean (ml):	**+**	p < 0.001
			Left lateral: 8.7, supine: 6.0		Considerable reduction of the upper airway volume in the supine position compared to the lateral.
**Arai et al.** [37] (2005) Japan	18 children (1–11 years) with OSA, scheduled for ENT surgery.	Observational study of airway obstruction in general anesthesia, in lateral and supine position, using stridor score.	Stridor score, median:	**+**	p < 0.05
			Lateral: 3, supine: 4		Supine position reduced the airway obstruction. (Addition of jaw thrust and/or chin lift reduced the obstruction further.)

**Table 4:** Studies reporting Respiratory Disturbance Index (RDI)

**Study** **(Year)**	**Patients**	**Study details**	**Results**	**Favors lateral**	**Notes**
**Country**					
***Adults with cervical spine cord injury (CSCI)***					
**McEvoy** ***et al.*** [42] (1995) Australia	42 adults with existing CSCI (46% of the identified candidates in a region)	Observational sleep study of RDI in supine vs. other sleeping positions.	RDI (events/h), mean:	**+**	p < 0.0005
			Non-supine sleeping positions: 15.3		No data for lateral position per se.
			Supine position: 23.6		
***Children with possible obstructive sleep apnea (OSA)***					
**Pereira ** ***et al.*** [44] (2005) USA	60 children (under 3 years), referred because of possible OSA	Observational data from previous sleep study.	RDI (events/h), mean:	**+**	p = 0.02
			Non-supine sleeping positions: 7.2		No data for lateral position per se.
			Supine position: 18.5		
***Adults with stroke***					
**Turkington** ***et al.*** [43] (2002) UK	120 stroke patients investigated more than 72 h after onset	Observational study of RDI in different sleeping positions.	RDI (events/h), mean:	**+**	p < 0.0001
			Left lateral position: 14; Right lateral: 12		Numbers for left and right lateral are not reported in text or table, but estimated from figure.
			Supine: 29

**Table 5:** Studies reporting AHI but not applicable for meta-analysis

**Study**	**Patients**	**Study details**	**Results**	**Favors lateral**	**Notes**
**(Year, country)**					
***Children with obstructive sleep apnea (OSA)***					
**Zhang** [47] (2007) China	45 children (3–13 years) with OSA	Observational study of AHI in lateral vs. supine sleeping positions, measured by PSG.	AHI (events/h), median:	**+**	Reports IQR, not SD.
			0 in left and right lateral position, 11.9 in supine-		P < 0.001 and p = 0.003, respectively.
**do Prado** [45] (2002) USA	80 children (1–10 years) with suspected OSA	Observational study of obstructive AHI in lateral vs. supine sleeping positions, measured by PSG.	Obstructive AHI (events/h), mean:	**?**	Does not report SD.
			7 in lateral positions, 8 in supine-		No significant difference.
**Nisbet** [50] (2014) Australia	76 children with Down syndrome (DS), 76 without DS	Observational study of AHI in DS, with matched controls.	AHI (events/h), median:		We report data from control group, as DS may be too indirect.
			REM sleep: 8.3 in non-supine positions, 17.8 in supine position	**+**	
					Reports IQR, not SD.
			Non-REM: 4.6 in non-supine positions, 5 in supine position.	**?**	
					In Non-REM sleep the difference is not clinically important.
***Adults with OSA***					
**Kim** [48] (2011) Korea	75 adults with OSA	Conference abstract of observational study of AHI in supine sleeping position vs. all other positions.	“This study confirms … that OSAS is position dependent in more than 50% of patients and non-supine position would lower the AHI…”	**+**	No data given, should be interpreted with caution.
**Sasai** [34] (2011) Japan	30 adults with moderate and severe OSA	Observational study of obstructive AHI in supine vs. all sleeping positions, measured by PSG.	AHI (events/h), mean:	**?**	Does not report AHI in lateral position per se.
			Moderate OSA: 27.0 in all positions, 27.5 in supine position.		
					In the severe group: p < 0.05, but not regarded as clinically significant difference.
			Severe OSA: 77.1 in all positions, 79.9 in supine position.		
**Li** [46] (2006) China	54 adults with OSA	Observational study of AHI in in lateral vs. supine sleeping positions, measured by PSG.	“…the overall AHI in supine position was higher than in lateral…”	**+**	Article in Chinese, only abstract in English, no data.
					p = 0.000
***Adults with stroke***					
**Svatikova** [49] (2011) USA	18 adults with stroke	Randomized crossover study of positional therapy for sleep apnea in stroke.	AHI (events/h), mean (no intervention):	**+**	Reports IQR, not SD.
					No p-value given.
			27 in non-supine positions, 49 in supine position.		

***Oxygen desaturation*** was reported in six studies (a total of 472 cases) [30,31,33-36] in various manners (e.g., the mean peripheral oxygen saturation [Sp0_2_], mean lowest Sp0_2_, and time between the desaturation episodes). In four of the comparisons (representing 217 persons), there was an indication of better oxygenation in the lateral position vs. the supine position. For the remaining comparisons, the differences were clinically insignificant (Table 2).

***The stridor score***, a four-step scale ranging from total obstruction to normal air passage (judged by stethoscopy) was reported in two studies [36,37]. The participants included 48 children under general anesthesia in the lateral and supine positions. Airway obstruction was reduced in the lateral vs. the supine positions. The addition of jaw thrust and/or chin lift further reduced the obstruction (Table 3).

***Upper airway resistance*** was reported in one study [38], in which there was a small difference between the two positions in favor of the lateral position (Table 3).

***The upper airway closing pressure*** was reported in two studies (n = 24) [39,40] as a measure of collapsibility. Both studies observed a lower collapsibility in the lateral position than in the supine position (Table 3).

***The inspiratory minute volume (MV)*** was reported in one study [38], and there was a small difference in favor of the lateral position (Table 3).

***The volume of the upper airway*** was reported in one study [41], wherein there was a greater volume in the lateral position (Table 3).

***The RDI*** (the number of episodes of apnea, hypopnea and respiratory-effort related arousals per hour) was reported in three studies [42-44]. Two of these studies included adults: one study included adults with cervical spine injuries, and the other included adults with stroke. The third study included children with possible obstructive sleep apnea. In these three studies, the investigators reported a statistically significant reduction in the RDI in the lateral position vs. the supine position (Table 4).

***The AHI*** (the number of episodes of apnea or hypopnea per hour) was reported in 27 studies. In seven of these studies [34,45-50], the AHI was incompletely reported, and, therefore, could not be included in the meta-analysis. However, four of the six studies showed a reduction of the AHI in the lateral position, indicating improved airway patency (Table 5).

Seventeen studies [31,35,51-65] of adults with sleep apnea, stroke, or undergoing surgery, with 26 comparisons, were suitable for meta-analysis (Figure 6). In all three groups, the lateral position significantly reduced the AHI compared to the supine position. In infants and small children [66-68], there was no significant difference between the two positions (Figure 6).

### Grading the evidence

Table 6 show the Summary of findings table for the comparison between the lateral and the supine position for patients with reduced consciousness. Full details are shown in the GRADE evidence profile (Additional file 3). The quality of the evidence was moderate for one of the four outcome comparisons. For the remainder, we rated the quality of evidence as low or very low.

## Discussion

In our systematic review of airway patency in unconscious trauma patients, we focused on the effect of placing the patient in the supine position vs. the lateral position. We did not identify any studies reporting mortality, morbidity or other, more indirect, outcome measures in trauma patients. One reason for the lack of such studies could be the associated logistical and ethical issues [69,70]. Another likely cause is that placing unconscious patients in the lateral position is considered to be an obvious solution and regarded as a truth (“textbook knowledge”) that does not require investigation. Thus, this practice may be regarded as a dogma, a strong belief based on experience more than scientific evidence. However, many EMS systems worldwide dictate the use of rigorous supine immobilization regimes in unconscious trauma patients [13,71,72]. We view this practice as an unsolved contradiction.

Due to the lack of specific studies in trauma patients, we decided to broaden the inclusion criteria to patients who had reduced level of consciousness from all causes. Five studies compared the supine airway patency in the awake vs. unconscious states [25-28]. One of these studies is the 1959 milestone publication by Peter Safar et al. [25]. In this study, the investigators anesthetized 80 elective surgery patients in the supine position and scored the airways as either open or partially or totally obstructed. In the neutral head position, 36% of the patients had total obstruction, while 54% had partial obstruction. These findings may have been considered to be proof that does not require further investigation. The findings in Safar’s study provide strong evidence that the supine position may endanger the airway in all unconscious patients. We see no reason to suspect that this conclusion is not true in unconscious trauma patients; on the contrary, trauma may further endanger the airway with factors such as bleeding from facial injuries and gastrointestinal regurgitation.

**Table 6:** Summary of Findings (GRADE): Lateral position compared to supine position for patients with reduced consciousness

**Outcomes**	**Illustrative comparative risks (95% CI)**		**№ of participants**	**Quality of the evidence**
	**Assumed risk**	**Corresponding risk**	**(Studies)**	**(GRADE)**
	**Supine position**	**Lateral position**		
**AHI - Adults with sleep apnea**	The median AHI (episodes/h) in the control group was **52.4**	The mean AHI (episodes/h) in the intervention group was **22.8 fewer** (29.1 fewer to16.6 fewer)	2780 (20 observational comparisons) ^1^	
**AHI - Adults before and after surgery**	The median AHI (episodes/h) in the control group was **26.0**	The mean AHI (episodes/h) in the intervention group was **10.4 fewer** (15.2 fewer to 5.6 fewer)	1448 (3 observational comparisons)	
**AHI - Patients with stroke/TIA**	The median AHI (episodes/h) in the control group was **23**	The mean AHI (episodes/h) in the intervention group was **13.9 fewer** (20.9 fewer to 6.8 fewer)	196 (2 observational studies)	
**AHI - Infants and small children**	The median AHI (episodes/h) in the control group was **2.5**	The mean AHI (episodes/h) in the intervention group was **0.74 more** (0.6 fewer to 2.08 more)	190 (9 observational comparisons)	

1. Three more studies were not included: Not sufficient data for analysis given.

2. Studies in which patients were their own controls.

3. In a number of the studies there was unclear bias regarding representativity, but internal validity was intact so we did not downgrade for this.

4. Unexplained heterogeneity regarding the size of effect, but a clear effect estimate in favor of the intervention. We upgraded for large effect.

5. Indirectness in population.

6. Small cumulative sample size, but clear benefit.

7. Unexplained heterogeneity regarding direction of effect, I^2^=90%, we downgraded for this uncertainty.

8. CI 95% includes both benefit and harm, but clinically insignificant difference.

In patients with reduced consciousness, we found evidence that the lateral position is better for securing an open airway than the supine position in a variety of settings. Our findings support the long-held recommendation to use a lateral position for all unconscious patients, including trauma. However, there are several caveats to the interpretation of our findings.

There are several limitations to the material available for this systematic review and meta-analysis. The first and foremost is the lack of direct endpoints and the absence of RCTs. The quality of evidence is lowered by indirectness in population. However, the effect size of the lateral position for improved airway patency (reduced AHI) in adults led to upgrading the quality of the evidence. During sleep, the difference between the two positions is likely to be greater with deeper levels of unconsciousness. We found considerable unexplained heterogeneity regarding the size of effect on AHI between the studies for adults; however, we did not downgrade for this variation.

Another limitation is that we did not address other concerns that were linked to the lateral positioning of trauma patients. One such concern is whether turning a patient with a cervical spine injury from the supine to the lateral position worsens the injury. We are in the process of addressing this question in a separate systematic review [18].

## Conclusions

In this systematic review, we did not identify any studies that investigated the supine position and loss of airway patency in trauma patients. However, we found that the supine position was associated with worse airway patency in patients with reduced levels of consciousness in a variety of settings. We also observed that the lateral position was associated with improved airway patency compared to the supine position. Although concerns other than airway patency may influence how the trauma patient is positioned, our systematic review provides evidence supporting the long-held recommendation to place the unconscious trauma patient in a lateral position.

## Consent

Written informed consent was obtained from the models for publication of the accompanying images.

## Additional files

Additional file 1:
**Search strategy.**
Additional file 2:
**Excluded full text articles.**
Additional file 3:
**GRADE evidence profile.**
