# Supplementary material for: Is the supine position associated with loss of airway patency in unconscious trauma patients? A systematic review and meta-analysis
Source: Scand J Trauma Resusc Emerg Med. 2015 Jul 1;23:50. doi: 10.1186/s13049-015-0116-0 (PMC4486423; doi:10.1186/s13049-015-0116-0)
Supplement: Additional file 1: — Search strategy. [file 13049_2015_116_MOESM1_ESM.docx]

# Appendix 1: Search strategy

*Search completed 27.01.2012, updated 30.06.2014*

*PICO question*

*Lying supine/on back when unconscious / with reduced level of consciousness / in coma /not awake, may this result in loss of (patent) airway or lead to hypoxia and /or hyperventilation?*

**Database: Ovid MEDLINE(R) <1946 to January Week 3 2012>**

**Search Strategy: 27.1.2012**

**Hits: 157**

1 exp Unconsciousness/ (30932)

2 Glasgow Coma Scale/ (5702)

3 unconscious*.tw. (7989)

4 (loss adj2 conscious*).tw. (3662)

5 (minimal adj2 conscious*).tw. (27)

6 coma*.tw. (24232)

7 pseudocoma*.tw. (7)

8 ((persistent* or transient* or permanent*) adj5 (unaware* or vegetativ* or state*)).tw. (5437)

9 (alter* adj2 mental adj2 status).tw. (1242)

10 syncope*.tw. (10020)

11 (glasgow adj2 scale).tw. (5377)

12 exp craniocerebral trauma/ (108562)

13 ((occipital or head or forehead or craniocerebral or cerebrocranial or cranial or parietal or temporal or frontal) adj2 (trauma* or wound*)).tw. (8747)

14 ((head or skull or craniocerebral or cerebrocranial or cranial) adj2 (injury or injuries)).tw. (19353)

15 Brain Injuries/ (36706)

16 ((brain or cortcial or cerebral or hemisphere) adj2 (injury or injuries or laceration* or trauma* or contusion*)).tw. (33065)

17 (tbi or tbis).tw. (9229)

18 ((trauma* or post-trauma* or post-concussive*) adj2 encephalopath*).tw. (134)

19 exp Sleep apnea syndromes/ (19632)

20 ((sleep or nocturnal) adj2 (apnea* or hypopnea*)).tw. (13707)

21 (sleep-disordered adj2 breathing).tw. (2778)

22 hypersomnia*.tw. (1130)

23 or/1-22 (213371)

24 supine position/ (4009)

25 patient positioning/ (692)

26 ((dorsal or supine or semisupine or semi supine or back) adj2 (posture* or position* or lying*)).tw. (9106)

27 (((back or spine) adj2 board*) or spineboard* or backboard*).tw. (50)

28 (vacuum adj mattress*).tw. (26)

29 or/24-28 (12066)

30 airway obstruction/ (14668)

31 ((airway* or airflow or respirator*) adj2 (obstruction* or loss or open or closure*)).tw. (16654)

32 (choke or choking).tw. (1270)

33 anoxia/ (47801)

34 brain hypoxia/ (6174)

35 (anoxemia* or anoxia* or hypoxia* or hypoxemia* or anoxic* or hypoxic*).tw. (94592)

36 hypercapnia/ (6754)

37 (hypercapnia* or hypercapnea*).tw. (7455)

38 Hypoventilation/ (1526)

39 hypoventilation*.tw. (3178)

40 Mortality/ (31366)

41 (mortality or mortalities or death*).tw. (709472)

42 morbidity/ (21140)

43 (morbidity or morbidities).tw. (194417)

44 glasgow outcome scale/ (949)

45 (glasgow adj outcome).tw. (2056)

46 or/30-45 (940803)

47 23 and 29 and 46 (157)

***************************

**Database: Embase <1980 to 2012 Week 03>**

**Search Strategy: 27.01.2012**

**Hits: 311**

--------------------------------------------------------------------------------

1 exp unconsciousness/ (58531)

2 Glasgow coma scale/ (9534)

3 unconscious*.tw. (10509)

4 (loss adj2 conscious*).tw. (4857)

5 (minimal adj2 conscious*).tw. (47)

6 coma*.tw. (29088)

7 pseudocoma*.tw. (8)

8 ((persistent* or transient* or permanent*) adj5 (unaware* or vegetativ* or state*)).tw. (6305)

9 (alter* adj2 mental adj2 status).tw. (1803)

10 syncope*.tw. (13321)

11 (glasgow adj2 scale).tw. (6909)

12 exp brain injury/ (93995)

13 head injury/ (32720)

14 ((occipital or head or forehead or craniocerebral or cerebrocranial or cranial or parietal or temporal or frontal) adj2 (trauma* or wound*)).tw. (10801)

15 ((head or skull or craniocerebral or cerebrocranial or cranial) adj2 (injury or injuries)).tw. (22718)

16 ((brain or cortial or cerebral or hemisphere) adj2 (injury or injuries or laceration* or trauma* or contusion*)).tw. (43925)

17 (tbi or tbis).tw. (13007)

18 ((trauma* or post-trauma* or post-concussive*) adj2 encephalopath*).tw. (173)

19 sleep apnea syndrome/ (28119)

20 ((sleep or nocturnal) adj2 (apnea* or hypopnea*)).tw. (18657)

21 (sleep-disordered adj2 breathing).tw. (3945)

22 hypersomnia*.tw. (1541)

23 or/1-22 (260895)

24 supine position/ (8708)

25 patient positioning/ (10621)

26 ((dorsal or supine or semisupine or semi supine or back) adj2 (posture* or position* or lying*)).tw. (11254)

27 (((back or spine) adj2 board*) or spineboard* or backboard*).tw. (67)

28 (vacuum adj mattress*).tw. (38)

29 or/24-28 (24899)

30 airway obstruction/ (20552)

31 ((airway* or airflow or respirator*) adj2 (obstruction* or loss or open or closure*)).tw. (19703)

32 (choke or choking).tw. (1646)

33 exp hypoxemia/ (91226)

34 brain hypoxia/ (8980)

35 (anoxemia* or anoxia* or hypoxia* or hypoxemia* or anoxic* or hypoxic*).tw. (112821)

36 hypercapnia/ (11315)

37 (hypercapnia* or hypercapnea*).tw. (8333)

38 hypoventilation/ (3575)

39 hypoventilation*.tw. (3825)

40 mortality/ (397347)

41 (mortality or mortalities or death*).tw. (875995)

42 morbidity/ (150677)

43 (morbidity or morbidities).tw. (253337)

44 glasgow outcome scale/ (1743)

45 (glasgow adj outcome).tw. (2757)

46 or/30-45 (1327578)

47 23 and 29 and 46 (311)

***************************

**ISI Web of Science**

**Dato: 27.01.2012**

**Hits: 472**

# 29 #28 AND #20 (471)

# 28 #27 OR #26 OR #25 OR #24 OR #23 OR #22 OR #21 (987 238)

# 27 TI=("glasgow outcome scale") OR TS=("glasgow outcome scale") (1 628)

# 26 TI=(mortality or mortalities or death* or morbidity or morbidities) OR TS=(mortality or mortalities or death* or morbidity or morbidities) (885 415)

# 25 TI=hypoventilation* OR TS=hypoventilation* (2 782)

# 24 TI=(hypercapnia* or hypercapnea*) OR TS=(hypercapnia* or hypercapnea*) (7 325)

# 23 TI=(anoxemia* or anoxia* or hypoxia* or hypoxemia*) OR TS=(anoxemia* or anoxia* or hypoxia* or hypoxemia*) (89 518)

# 22 TI=(choke or choking) OR TS=(choke or choking) (3 156)

# 21 TI=((airway* or airflow or respirator*) NEAR/2 (obstruction* or loss or open or closure*)) OR TS=((airway* or airflow or respirator*) NEAR/2 (obstruction* or loss or open or closure*)) (15 775)

# 20 #19 AND #15 (2 940)

# 19 #18 OR #17 OR #16 (299 240)

# 18 TI=(vacuum NEAR/2 mattress*) OR TS=(vacuum NEAR/2 mattress*) (27)

# 17 TI=(((back or spine) NEAR/2 board*) or spineboard* or backboard*) OR TS=(((back or spine) NEAR/2 board*) or spineboard* or backboard*) (125)

# 16 TI=(dorsal or supine or semisupine or semi supine or back) OR TS=(dorsal or ) (299 183)

# 15 #14 OR #13 OR #12 OR #11 OR #10 OR #9 OR #8 OR #7 OR #6 OR #5 OR #4 OR #3 OR #2 OR #1 (147 238)

# 14 TI=hypersomnia* OR TS=hypersomnia* (1 082)

# 13 TI=(sleep-disordered NEAR/2 breathing) OR TS=(sleep-disordered NEAR/2 breathing) (3 342)

# 12 TI=((sleep or nocturnal) NEAR/2 (apnea* or hypopnea*)) OR TS=((sleep or nocturnal) NEAR/2 (apnea* or hypopnea*)) (18 779)

# 11 TI=((trauma* or post-trauma* or post-concussive*) NEAR/2 encephalopath*) or TS=((trauma* or post-trauma* or post-concussive*) NEAR/2 encephalopath*) (129)

# 10 TI=(tbi or tbis) or TS=(tbi or tbis) (9 955)

# 9 TI=((brain or cortial or cerebral or hemisphere) NEAR/2 (injury or injuries or laceration* or trauma* or contusion*)) OR TS=((brain or cortial or cerebral or hemisphere) NEAR/2 (injury or injuries or laceration* or trauma* or contusion*)) (49 579)

# 8 TI=((head or skull or craniocerebral or cerebrocranial or cranial) NEAR/2 (injury or injuries)) OR TS=((head or skull or craniocerebral or cerebrocranial or cranial) NEAR/2 (injury or injuries)) (25 995)

# 7 TI=((occipital or head or forehead or craniocerebral or cerebrocranial or cranial or parietal or temporal or frontal) NEAR/2 (trauma* or wound*)) or TS=((occipital or head or forehead or craniocerebral or cerebrocranial or cranial or parietal or temporal or frontal) NEAR/2 (trauma* or wound*)) (9 004)

# 6 TI=((persistent* or transient* or permanent*) NEAR/3 (unaware* or vegetativ* or state*)) OR TS=((persistent* or transient* or permanent*) NEAR/3 (unaware* or vegetativ* or state*)) (13 349)

# 5 TI=(minimal* NEAR/2 conscious*) or TS=(minimal* NEAR/2 conscious*) (524)

# 4 TI=(loss NEAR/2 conscious*) or TS=(loss NEAR/2 conscious*) (2 667)

# 3 TI=(coma* or pseudocoma* or syncope*) OR TS=(coma* or pseudocoma* or syncope*) (34 828)

# 2 TI=("glasgow coma scale") OR TS=("glasgow coma scale") (3 742)

# 1 TS=unconscious* OR TI=unconscious* (10 267)

**Cocharane Library**

**Dato: 27.01.2012**

**Hits: 43 (Other Review)**

#1 MeSH descriptor Unconsciousness explode all trees (525)

#2 MeSH descriptor Glasgow Coma Scale explode all trees (296)

#3 unconscious*:ti,ab,kw (367)

#4 (loss NEAR/3 conscious*):ti,ab,kw (247)

#5 (minimal NEAR/3 conscious*):ti,ab,kw (7)

#6 (coma* or pseudocoma*):ti,ab,kw (1212)

#7 ((persistent* or transient* or permanent*) NEAR/5 (unaware* or vegetativ* or state*)):ti,ab,kw (96)

#8 (alter* NEAR/3 mental NEAR/3 status):ti,ab,kw (23)

#9 syncope*:ti,ab,kw (578)

#10 (glasgow NEAR/2 scale):ti,ab,kw (709)

#11 MeSH descriptor Craniocerebral Trauma explode all trees (1563)

#12 ((occipital or head or forehead or craniocerebral or cerebrocranial or cranial or parietal or temporal or frontal) NEAR/2 (trauma* or wound*)):ti,ab,kw (450)

#13 ((head or skull or craniocerebral or cerebrocranial or cranial) NEAR/2 (injury or injuries)):ti,ab,kw (810)

#14 MeSH descriptor Brain Injuries explode all trees (854)

#15 ((brain or cortial or cerebral or hemisphere) NEAR/2 (injury or injuries or laceration* or trauma* or contusion*)):ti,ab,kw (1527)

#16 (tbi or tbis):ti,ab,kw (451)

#17 ((trauma* or post-trauma* or post-concussive*) NEAR/2 encephalopath*):ti,ab,kw (5)

#18 MeSH descriptor Sleep Apnea Syndromes explode all trees (1030)

#19 ((sleep or nocturnal) NEAR/2 (apnea* or hypopnea*)):ti,ab,kw (1788)

#20 (sleep-disordered NEAR/2 breathing):ti,ab,kw (189)

#21 hypersomnia*:ti,ab,kw (65)

#22 (#1 OR #2 OR #3 OR #4 OR #5 OR #6 OR #7 OR #8 OR #9 OR #10 OR #11 OR #12 OR #13 OR #14 OR #15 OR #16 OR #17 OR #18 OR #19 OR #20 OR #21) (6833)

#23 MeSH descriptor Supine Position explode all trees (450)

#24 MeSH descriptor Patient Positioning explode all trees (39)

#25 ((dorsal or supine or semisupine or semi supine or back) NEAR/2 (posture* or position*)):ti,ab,kw (1518)

#26 (((back or spine) NEAR/2 board*) or spineboard* or backboard*):ti,ab,kw (25)

#27 (vacuum NEAR mattress*):ti,ab,kw (6)

#28 (#23 OR #24 OR #25 OR #26 OR #27) (1554)

#29 (#22 AND #28) (54)

**Cinahl**

**Dato: 27.01.2012**

**Hits: 311**

S29 S22 and S28 (311)

S28 S23 or S24 or S25 or S26 or S27 (5954)

S27 TI (vacuum N2 mattress*) OR AB (vacuum N2 mattress*) (9)

S26 TI ( (((back or spine) N2 board*) or spineboard* or backboard*) ) OR AB ( (((back or spine) N2 board*) or spineboard* or backboard*) ) (103)

S25 TI ((dorsal or supine or semisupine or semi supine or back) N3 (posture* or position* or lying*)) OR AB ((dorsal or supine or semisupine or semi supine or back) N3 (posture* or position* or lying*)) (1377)

S24 (MH "Patient Positioning") (4173)

S23 (MH "Supine Position") (1089)

S22 S1 or S2 or S3 or S4 or S5 or S6 or S7 or S8 or S9 or S10 or S11 or S12 or S13 or S14 or S15 or S16 or S17 or S18 or S19 or S20 or S21 (32488)

S21 TI hypersomnia* OR AB hypersomnia* (60)

S20 TI (sleep-disordered N2 breathing) OR AB (sleep-disordered N2 breathing) (659)

S19 TI ( ((sleep or nocturnal) N2 (apnea* or hypopnea*)) ) OR AB ( ((sleep or nocturnal) N2 (apnea* or hypopnea*)) ) (2702)

S18 (MH "Sleep Apnea Syndromes+") (4199)

S17 TI ( ((trauma* or post-trauma* or post-concussive*) N2 encephalopath*) ) OR AB ( ((trauma* or post-trauma* or post-concussive*) N2 encephalopath*) ) (20)

S16 TI ( tbi or tbis ) OR AB ( tbi or tbis ) (2720)

S15 TI ( ((brain or cortial or cerebral or hemisphere) N2 (injury or injuries or laceration* or trauma* or contusion*)) ) OR AB ( ((brain or cortial or cerebral or hemisphere) N2 (injury or injuries or laceration* or trauma* or contusion*)) ) (8814)

S14 TI ( ((head or skull or craniocerebral or cerebrocranial or cranial) N2 (injury or injuries)) ) OR AB ( ((head or skull or craniocerebral or cerebrocranial or cranial) N2 (injury or injuries)) ) (3505)

S13 TI ( ((occipital or head or forehead or craniocerebral or cerebrocranial or cranial or parietal or temporal or frontal) N2 (trauma* or wound*)) ) OR AB ( ((occipital or head or forehead or craniocerebral or cerebrocranial or cranial or parietal or temporal or frontal) N2 (trauma* or wound*)) ) (1320)

S12 (MH "Head Injuries+") (17205)

S11 TI glasgow N2 scale OR AB glasgow N2 scale (1320)

S10 TI syncope* OR AB syncope* (1775)

S9 TI alter* N3 mental N3 status OR AB alter* N3 mental N3 status (368)

S8 TI ( ((persistent* or transient* or permanent*) N5 (unaware* or vegetativ* or state*)) ) OR AB ( ((persistent* or transient* or permanent*) N5 (unaware* or vegetativ* or state*)) ) (408)

S7 TI ( coma* or pseudocoma* ) OR AB ( coma or pseudocoma* ) (2485)

S6 TI ( ((loss or minimal) N2 conscious*) ) OR AB ( ((loss or minimal) N2 conscious*) ) (489)

S5 TI unconscious* OR AB unconscious* (961)

S4 (MH "Coma") (1040)

S3 (MH "Comatose Patients") (137)

S2 (MH "Glasgow Coma Scale") (3191)

S1 (MH "Unconsciousness+")
