# Supplementary material for: Is the supine position associated with loss of airway patency in unconscious trauma patients? A systematic review and meta-analysis
Source: Scand J Trauma Resusc Emerg Med. 2015 Jul 1;23:50. doi: 10.1186/s13049-015-0116-0 (PMC4486423; doi:10.1186/s13049-015-0116-0)
Supplement: Additional file 2: — Excluded full text articles. [file 13049_2015_116_MOESM2_ESM.docx]

**Appendix 2: Excluded full text articles**

| Study | Reason for exclusion |
| --- | --- |
| Adams JP, Murphy PG: **Obesity in anaesthesia and intensive care.** *British Journal of Anaesthesia* 2000, **85:**91-108. | Review article, contains no data set. Initially included because of possible references. |
| Agro F, Salvinelli F, Casale M, Gherardi S: **Difficulty in airway management during sedation of patients affected by obstructive sleep apnea.** *Canadian Journal of Anesthesia* 2004, **51:**279. | Letter to editor, case reports, no relevant data. |
| Arai YC, Nakayama M, Kato N, Wakao Y, Ito H, Komatsu T: **The effects of jaw thrust and the lateral position on heart rate variability in anesthetized children with obstructive sleep apnea syndrome.** *Anesthesia and Analgesia* 2007, 104: 1352-1355 | Only baseline data relevant, but these are identical to included study. |
| Augustine JJ: **Unconventional patient positioning 236.** *Emergency Medical Services* 1998, **27:**60. | No study. Initially included because of possible references. |
| **Basic Life Support: Airway. ARC and NZRC Guideline 2010 6.** *Emergency Medicine Australasia* 2011, **23:**248-251. | Guideline article, no study. Initially included because of possible references. |
| **Basic Life Support: Unconsciousness. ARC and NZRC Guideline 2010 5.** *Emergency Medicine Australasia* 2011, **23:**246-247. | Guideline article, no study. Initially included because of possible references. |
| Christie RJ: **Therapeutic positioning of the multiply-injured trauma patient in ICU 76.** *British Journal of Nursing (BJN)* 2008, **17:**638-642. | No study. Initially included because of possible references. |
| Crewdson K, Nolan JP: **Management of the trauma airway.** *Trauma* 2011, **13:**221-232. | Review, no study. Initially included because of possible references. |
| Dunham CM, Brocker BP, Collier BD, Gemmel DJ: **Risks associated with magnetic resonance imaging and cervical collar in comatose, blunt trauma patients with negative comprehensive cervical spine computed tomography and no apparent spinal deficit.** *Critical Care (London, England)* 2008, **12:**R89. | No relevant outcome reported for lateral vs. supine position, nor awake vs. asleep in supine position. |
| Ferretti A, Giampiccolo P, Cavalli A, Milic-Emili J, Tantucci C: **Expiratory flow limitation and orthopnea in massively obese subjects 195.** *Chest* 2001, **119:**1401-1408. | Awake patients. |
| Fouke JM, Strohl KP: **Effect of position and lung volume on upper airway geometry.** *Journal of Applied Physiology* 1987, **63:**375-380. | Awake patients |
| Fryman L, Murray L: **Managing acute head trauma in a crowded emergency department 89.** *JEN: Journal of Emergency Nursing* 2007, **33:**208-213. | No study. Initially included because of possible references. |
| Guardiola J, Yu J, Hasan N, Fletcher EC: **Evening and morning blood gases in patients with obstructive sleep apnea.** *Sleep Medicine* 2004, **5:**489-493. | No relevant outcomes. Initially included because of possible references. |
| Gunn BD, Eizenberg N, Silberstein M, McMeeken JM, Tully EA, Stillman BC, Brown DJ, Gutteridge GA: **How should an unconscious person with a suspected neck injury be positioned?** *Prehospital and Disaster Medicine* 1995, **10:**239-244. | Awake patients. |
| Haines J: **Positioning an unconscious patient with suspected neck injury 259.** *JEMS: Journal of Emergency Medical Services* 1996, **21:**85-85. | Descriptive, no study. Initially included because of possible references. |
| Hjalmarsen A, Hykkerud DL: **Severe nocturnal hypoxaemia in geriatric inpatients 66.** *Age & Ageing* 2008, **37:**526-529. | No relevant outcome reported for lateral vs. supine position, nor awake vs. asleep in supine position. |
| Hodgman JE: **Effect on sleep position on apnea and bradycardia in high-risk infants 175.** *Journal of Perinatology* 2002, **22:**163-164. | No study. Initially included because of possible references. |
| Iida S, Kogo M, Ishii S, Kohara H, Matsuya T: **Changes of arterial oxygen saturation (SpO(2)) following push-back operation.** *International Journal of Oral and Maxillofacial Surgery* 1998, **27:**425-427. | Outcome is not reported for lateral vs. supine position, nor awake vs. asleep in supine position. |
| Ingman T, Nieminen T, Hurmerinta K: **Cephalometric comparison of pharyngeal changes in subjects with upper airway resistance syndrome or obstructive sleep apnoea in upright and supine positions.** *European Journal of Orthodontics* 2004, **26:**321-326. | Awake patients. |
| Jung DG, Cho HY, Grunstein RR, Yee B: **Predictive value of Kushida index and acoustic pharyngometry for the evaluation of upper airway in subjects with or without obstructive sleep apnea.** *Journal of Korean medical science* 2004, **19:**662-667. | No relevant outcome reported for lateral vs. supine position, nor awake vs. asleep in supine position. |
| Kahn A, Groswasser J, Sottiaux M, Rebuffat E, Franco P, Dramaix M: **Prone or supine body position and sleep characteristics in infants.** *Pediatrics* 1993, **91:**1112-1115. | No relevant outcome is reported for lateral vs. supine position, nor awake vs. asleep in supine position. |
| Kim JW, Yoon IY, Chung S, Lee CH, Moon SJ, Yun PY: **Comparison between tongue base and soft palate obstruction in obstructive sleep apnea.** *Acta Oto-Laryngologica* 2009, **129:**855-861. | No relevant outcome reported for lateral vs. supine position, nor awake vs. asleep in supine position. |
| Langan Y, Nashef L, Sander JWAS: **Sudden unexpected death in epilepsy: A series of witnessed deaths.** *Journal of Neurology Neurosurgery and Psychiatry* 2000, **68:**211-213. | Case review, outcome is not reported for lateral vs. supine position. |
| Ledwith M, Bloom S, Maloney-Wilensky E, Coyle B, Polomano R, D: **Effect of body position on cerebral oxygenation and physiologic parameters in patients with acute neurological conditions 19.** *Journal of Neuroscience Nursing* 2010, **42:**280-287. | No relevant outcome reported for lateral vs. supine position, nor awake vs. asleep in supine position. |
| Levendowski DJ, Berka C, Popovic D, Scarfeo D, Zavora T, Westbrook P: **Impact of age and position on severity of gender-specific sleep disordered breathing.** *Sleep* 2009, Conference: 23rd Annual Meeting of the Associated Professional Sleep Societies Seattle, WA United States. Conference Start: 20090606 Conference End: 20090611. Conference Publication**:** A214. | No relevant outcome reported for lateral vs. supine position, nor awake vs. asleep in supine position. |
| Maasilta P, Bachour A, Teramo K, Polo O, Laitinen LA: **Sleep-related disordered breathing during pregnancy in obese women 183.** *Chest* 2001, **120:**1448-1454. | No relevant outcome is reported for lateral vs. supine position, nor awake vs. asleep in supine position. |
| Mador MJ, Kufel TJ, Magalang UJ, Rajesh SK, Watwe V, Grant BJB: **Prevalence of positional sleep apnea in patients undergoing polysomnography 113.** *Chest* 2005, **128:**2130-2137. | No relevant outcome reported for lateral vs. supine position, nor awake vs. asleep in supine position. |
| Marklew A: **Body positioning and its effect on oxygenation--a literature review.** *Nurs Crit Care* 2006, **11:**16-22. | No study. Initially included because of possible references. |
| Mee LYS, Bee WH: **A comparison study on nurses' and therapists' perception on the positioning of stroke patients in Singapore General Hospital 88.** *International Journal of Nursing Practice* 2007, **13:**209-221. | No relevant outcome reported for lateral vs. supine position, nor awake vs. asleep in supine position. |
| Miura C, Hida W, Miki H, Kikuchi Y, Chonan T, Takishima T: **Effects of posture on flow-volume curves during normocapnia and hypercapnia in patients with obstructive sleep apnoea.** *Thorax* 1992, **47:**524-528. | Awake patients. |
| Morikawa S, Safar P, Decarlo J: **Influence of the headjaw position upon upper airway patency.** *Anesthesiology* 1961, **22:**265-270. | Only outcome measure was AP diameter of the upper airway, not cross sectional area, too indirect |
| Morris M: **Transport considerations for the head-injured patient: are we contributing to secondary injury? 286.** *Journal of Air Medical Transport* 1992, **11:**9-13. | Review article, no study. Initially included because of possible references. |
| Nelson JA, Loredo JS, Acosta JA: **The obesity-hypoventilation syndrome and respiratory failure in the acute trauma patient.** *Journal of Emergency Medicine* 2011, **40:**e67-e69. | No study. Initially included because of possible references. |
| Nightingale P: **The effect of positioning on the severity of sleep apnea syndrome and its relevance in the avoidance of hypoxia in acute stroke 137.** *Physical Therapy Reviews* 2004, **9:**161-172. | Systematic review. Initially included because of possible references. |
| Oksenberg A, Khamaysi I, Silverberg DS: **Apnoea characteristics across the night in severe obstructive sleep apnoea: influence of body posture.** *Eur Respir J* 2001, 18: 340-6 | Patient data identical to other study included [32] |
| Oksenberg A, Silverberg DS, Arons E, Radwan H: **Positional vs nonpositional obstructive sleep apnea patients: anthropomorphic, nocturnal polysomnographic, and multiple sleep latency test data.** *Chest* 1997, **112:**629-639. | Outcome is not reported for lateral vs. supine position, nor awake vs. asleep in supine position. |
| Oksenberg A, Silverberg DS: **Avoiding the supine posture during sleep for patients with mild obstructive sleep apnea... Am J Respir Crit Care Med. 2009 Feb 15;179(4):320-7 44.** *American Journal of Respiratory & Critical Care Medicine* 2009, **180:**101-102. | No study. Initially included because of possible references. |
| Ono T, Otsuka R, Kuroda T, Honda E, Sasaki T: **Effects of head and body position on two- and three-dimensional configurations of the upper airway.** *Journal of Dental Research* 2000, **79:**1879-1884. | Awake patients |
| Pirila K, Tahvanainen P, Huggare J, Nieminen P, Lopponen H: **Sleeping positions and dental arch dimensions in children with suspected obstructive sleep apnea syndrome.** *European Journal of Oral Sciences* 1995, **103:**285-291. | Outcome is not reported for lateral vs. supine position, nor awake vs. asleep in supine position. |
| Ponsonby AL, Dwyer T, Couper D: **Sleeping position, infant apnea, and cyanosis: a population-based study.** *Pediatrics* 1997, **99:**E3. | Outcome measures too indirect |
| Price AM, Collins TJ, Gallagher A: **Nursing care of the acute head injury: a review of the evidence 159.** *Nursing in Critical Care* 2003, **8:**126-133. | No study. Initially included because of possible references. |
| Reber A: **Airway management in sedated patients.** *Anaesthesist* 2011, **60:**259-264. | Review, no study. Initially included because of possible references. |
| Richard W, Kox D, Den HC, Laman M, Van TH, De VN: **The role of sleep position in obstructive sleep apnea syndrome.** *European Archives of Oto-Rhino-Laryngology* 2006, **263:**946-950. | No relevant outcome reported for lateral vs. supine position, nor awake vs. asleep in supine position. |
| Safar P: **From control of airway and breathing to cardiopulmonary-cerebral resuscitation.** *Anesthesiology* 2001, **95:**789-791. | Historical article. Original study is included. |
| Saigusa H, Suzuki M, Higurashi N, Kodera K: **Three-dimensional morphological analyses of positional dependence in patients with obstructive sleep apnea syndrome 51.** *Anesthesiology* 2009, **110:**885-890. | Awake patients. |
| Satoh M, Hida W, Chonan T, Okabe S, Miki H, Taguchi O, Kikuchi Y, Takishima T: **Effects of posture on carbon dioxide responsiveness in patients with obstructive sleep apnoea.** *Thorax* 1993, **48:**537-541. | Outcome is not reported for lateral vs. supine position, nor awake vs. asleep in supine position. |
| Scali VJ, Ferko JG, III, Dobay K, Becher J: **Handling head injuries 297.** *Emergency* 1989, **21:**22. | Review article, no study. Initially included because of possible references. |
| Schnoor J, Ilgner J, Hein M, Westhofen M, Rossaint R: **Perioperative management of patients with obstructive sleep apnoea.** *Anaesthesist* 2009, **58:**189-198. | Review, no study. Initially included because of possible references. |
| Schumann R: **Anaesthesia for bariatric surgery.** *Best Practice and Research: Clinical Anaesthesiology* 2011, **25:**83-93. | Review, no study. Initially included because of possible references. |
| Sharp JT, Druz WS, Kondragunta VR: **Diaphragmatic responses to body position changes in obese patients with obstructive sleep apnea.** *American Review of Respiratory Disease* 1986, **133:**32-37. | Outcome is not reported for lateral vs. supine position, nor awake vs. asleep in supine position. |
| Sivarajan M, Joy JV: **Effects of general anesthesia and paralysis on upper airway changes due to head position in humans.** *Anesthesiology:* 1996, 85: 787-793 | Reports AP distance in the upper airway only, not cross-sectional area, too indirect. |
| Tyson SF, Nightingale P: **The effects of position on oxygen saturation in acute stroke: a systematic review.** *Clinical Rehabilitation* 2004, **18:**863-871. | Systematic review. References searched for eligible studies. |
| von Ungern-Sternberg BS, Erb TO, Frei FJ: **Management of the upper airway in spontaneously breathing children. A challenge for the anaesthetist.** *Anaesthesist* 2006, **55:**164-170. | No study. Initially included because of possible references. |
| Winkelman C: **Effect of backrest position on intracranial and cerebral perfusion pressures in traumatically brain-injured adults 200.** *American Journal of Critical Care* 2000, **9:**373-382. | No relevant outcome for lateral vs. supine position. |
| Yildirim N, Fitzpatrick MF, Whyte KF, Jalleh R, Wightman AJA, Douglas NJ: **The effect of posture on upper airway dimensions in normal subjects and in patients with the sleep apnea/hypopnea syndrome.** *American Review of Respiratory Disease* 1991, **144:**845-847. | Awake patients. |
| Zeng B, Ng AT, Darendeliler MA, Petocz P, Cistulli PA: **Use of flow-volume curves to predict oral appliance treatment outcome in obstructive sleep apnea 94.** *American Journal of Respiratory & Critical Care Medicine* 2007, **175:**726-730. | No relevant outcome reported for lateral vs. supine position, nor awake vs. asleep in supine position. |
| McEvoy RD, Sharp DJ, Thornton AT: **The effects of posture on obstructive sleep apnea.** *Am Rev Respir Dis* 1986, **133:**662-666. | Asleep patients in supine vs. 60 degrees elevation, not asleep supine vs. awake supine. |
| Seet E, Chung F: **Management of sleep apnea in adults - functional algorithms for the perioperative period: Continuing Professional Development.** *Can J Anaesth* 2010, **57:**849-864. | Review article, contains no data set. Initially included because of possible references. |
| Abdullah B, Rajet KAM, Abd Hamid SS, Mohammad WMZW: **A videoendoscopic evaluation of the upper airway in South East Asian adults with obstructive sleep apnea.** *Sleep and Breathing* 2011, **15:**747-754. | Awake patients. |
| Bharadwaj R, Ravikumar A, Krishnaswamy NR: **Evaluation of craniofacial morphology in patients with obstructive sleep apnea using lateral cephalometry and dynamic MRI.** *Indian J Dent Res* 2011, **22:**739-748. | No relevant outcome reported for lateral vs. supine position, nor awake vs. asleep in supine position. |
| Jordan AS, Cori J, Rainbird EM, Trinder JA, O'Donoghue FJ, Rochford P: **Lung volume during wake and sleep in Obstructive Sleep Apnea (OSA).** *Am J Respir Crit Care Med* 2011, **183 (1 MeetingAbstracts)**. | No relevant outcome reported for lateral vs. supine position, nor awake vs. asleep in supine position. |
| Baltzan MA, Scott AS, Wolkove N: **Unilateral Hemidiaphragm Weakness Is Associated with Positional Hypoxemia in REM Sleep.** *Journal of Clinical Sleep Medicine* 2012, **8:**51-58. | Variable is affected lung up or down, not relevant. |
| Collopy KT, Kivlehan S, Snyder SR: **HOW OBESITY Impacts Patient Health and EMS.** *EMS World* 2012, **41:**40-49. | Review article, contains no data set. Initially included because of possible references. |
| de Dios JAA, Brass SD: **New and Unconventional Treatments for Obstructive Sleep Apnea.** *Neurotherapeutics* 2012, **9:**702-709. | Review article, contains no data set. Initially included because of possible references. |
| Gammon BT, Ricker KF: **An Evidence-Based Checklist for the Postoperative Management of Obstructive Sleep Apnea.** *J Perianesth Nurs* 2012, **27:**316-322. | Review article, contains no data set. Initially included because of possible references. |
| Hankins D: **Time to Abandon the GCS in Prehospital Care?** *Air Med J* 2012, **31:**70-71. | No study. Initially included because of possible references. |
| Taranto Montemurro L, Bettinzoli M, Corda L, Redolfi S, Novali M, Braghini A, Tantucci C: **Influence of upper airway size on volume exhaled under negative pressure during evaluation of upper airway collapsibility.** *Sleep & Breathing* 2012, **16:**399-404. | No relevant outcome reported for lateral vs. supine position, nor awake vs. asleep in supine position. |
| Toh ST, Lin CH, Guilleminault C: **Usage of four-phase high-resolution rhinomanometry and measurement of nasal resistance in sleep-disordered breathing.** *Laryngoscope* 2012, **122:**2343-2349. | No relevant outcome reported for lateral vs. supine position, nor awake vs. asleep in supine position. |
| Dalesio NM, Stierer TL, Schwartz AR: **Upper airway physiology in sleep and anesthesia.** *Sleep Medicine Clinics* 2013, **8:**29-41. | Review article, contains no data set. Initially included because of possible references. |
| Damkliang J, Considine J, Kent B: **Thai emergency nurses’ management of patients with severe traumatic brain injury: Comparison of knowledge and clinical management with best available evidence.** *Australasian Emergency Nursing Journal* 2013, **16:**127-135. | No relevant outcome reported for lateral vs. supine position, nor awake vs. asleep in supine position. |
| Fuller DD, Lee KZ, Tester NJ: **The impact of spinal cord injury on breathing during sleep.** *Respiratory Physiology and Neurobiology* 2013, **188:**344-354. | Review article, contains no data set. Initially included because of possible references. |
| Isono S: **Pathophysiologic considerations of perioperative respiratory managements of obese patients with obstructive sleep apnea.** *Sleep Medicine Clinics* 2013, **8:**59-64. | Editorial, no study data. Initially included because of possible references. |
| Jaimchariyatam N, Dweik RA, Kaw R, Aboussouan LS: **Polysomnographic determinants of nocturnal hypercapnia in patients with sleep apnea.** *Journal of Clinical Sleep Medicine* 2013, **9:**209-215. | No relevant outcome reported for lateral vs. supine position, nor awake vs. asleep in supine position. |
| Koo SK, Choi JW, Myung NS, Lee HJ, Kim YJ: **Analysis of obstruction site in obstructive sleep apnea syndrome patients by drug induced sleep endoscopy.** *American Journal of Otolaryngology - Head and Neck Medicine and Surgery* 2013, **34:**626-630. | No relevant outcome reported for lateral vs. supine position, nor awake vs. asleep in supine position. |
| Matthews L, Fortier N: **The Rematee Bumper Belt® positional therapy device for snoring and obstructive sleep apnea: Positional effectiveness in healthy subjects.** *Canadian Journal of Respiratory Therapy* 2013, **49:**11-14. | No relevant outcome reported for lateral vs. supine position, nor awake vs. asleep in supine position. |
| National Association of EMSP, American College of Surgeons Committee on T: **EMS Spinal Precautions and the Use of the Long Backboard.** *Prehosp Emerg Care* 2013, **17:**392-393. | Guideline article, no study data. Initially included because of possible references. |
| Todea D, Todor I, Coman A: **Relationship of arterial hypertension and obstructive sleep apnea syndrome.** *Sleep Medicine* 2013, **14:**e100-e101. | No relevant outcome reported for lateral vs. supine position, nor awake vs. asleep in supine position. |
| Yaghmaei M, Ejlali M, Nikzad S, Sayyedi A, Shafaeifard S, Pourdanesh F: **General anesthesia in orthognathic surgeries: does it affect horizontal jaw relations?** *Journal of Oral & Maxillofacial Surgery (02782391)* 2013, **71:**1752-1756. | No relevant outcome reported for lateral vs. supine position, nor awake vs. asleep in supine position. |
| Adar T, Shteingart S, Katz D, Goldin E, Paz K: **Significant temporal association between gastroesophageal acid reflux events and obstructive sleep apnea. Results of the pilot swap study-synchronous wireless ph-metery and ambulatory polysomnography.** *Gastroenterology* 2014, **1):**S-857. | No relevant outcome reported for lateral vs. supine position, nor awake vs. asleep in supine position. |
| Dunham CM, Hileman BM, Hutchinson AE, Chance EA, Huang GS: **Perioperative hypoxemia is common with horizontal positioning during general anesthesia and is associated with major adverse outcomes: A retrospective study of consecutive patients.** *BMC Anesthesiology* 2014, **14**. | No relevant outcome reported for lateral vs. supine position, nor awake vs. asleep in supine position. |
| Lambert AA, Parker AM, Moon KK: **High-dose N-acetylcysteine in chronic obstructive pulmonary disease, prone positioning in acute respiratory distress syndrome, and continuous positive airway pressure and exhaled nitric oxide in obstructive sleep apnea.** *Am J Respir Crit Care Med* 2014, **189:**223-224. | Summary of three different studies. No relevant data. |
| Lump D: **MANAGING PATIENTS WITH SEVERE traumatic brain injury.** *Nursing (Lond)* 2014, **44:**30-38. | Review article, contains no data set. Initially included because of possible references. |
| Morong S, Hermsen B, De Vries N: **Sleep-disordered breathing in pregnancy: A review of the physiology and potential role for positional therapy.** *Sleep and Breathing* 2014, **18:**31-37. | Review article, contains no data set. Initially included because of possible references. |
| Oksenberg A, Gadoth N: **Are we missing a simple treatment for most adult sleep apnea patients? The avoidance of the supine sleep position.** *J Sleep Res* 2014, **23:**204-210. | Editorial, no study data. Initially included because of possible references. |
| Sankari A, Bascom A, Oomman S, Badr MS: **Sleep Disordered Breathing in Chronic Spinal Cord Injury.** *Journal of Clinical Sleep Medicine* 2014, **10:**65-72. | No relevant outcome reported for lateral vs. supine position, nor awake vs. asleep in supine position. |
