# Supplementary material for: Is the supine position associated with loss of airway patency in unconscious trauma patients? A systematic review and meta-analysis
Source: Scand J Trauma Resusc Emerg Med. 2015 Jul 1;23:50. doi: 10.1186/s13049-015-0116-0 (PMC4486423; doi:10.1186/s13049-015-0116-0)
Supplement: Additional file 3: — GRADE evidence profile. [file 13049_2015_116_MOESM3_ESM.docx]

**Appendix 3: GRADE evidence profile**

| **Quality assessment** | | | | | | | **№ of patients** | | **Effect** | | **Quality** | **Importance** |
| --- | --- | --- | --- | --- | --- | --- | --- | --- | --- | --- | --- | --- |
| **№ of studies** | **Study design** | **Risk of bias** | **Inconsistency** | **Indirectness** | **Imprecision** | **Other considerations** | **Lateral position** | **Supine position** | **Relative**  **(95% CI)** | **Absolute**  **(95% CI)** |  |  |
| ***AHI (episodes/h) - Adults with sleep apnea*** | | | | | | | | | | | | |
| 20 | observational comparisons ^1^ | not serious ^2,3^ | not serious ^4^ | not serious ^5^ | not serious | strong association | 1411 | 1369 | - | MD **22.8 fewer**  (29.1 fewer to 16.6 fewer) | ⨁⨁⨁◯  MODERATE | Important |
| ***AHI (episodes/h) – Adults before and after surgery*** | | | | | | | | | | | | |
| 3 | observational comparisons | not serious ^2^ | not serious | not serious ^5^ | not serious | none | 724 | 724 | - | MD **10.4 fewer**  (15.2 fewer to 5.6 fewer) | ⨁⨁◯◯  LOW | Important |
| ***AHI (episodes/h) - Patients with stroke/TIA*** | | | | | | | | | | | | |
| 2 | observational studies | not serious ^2^ | not serious | not serious ^5^ | not serious ^6^ | none | 98 | 98 | - | MD **13.9 fewer**  (20.9 fewer to 6.8 fewer) | ⨁⨁◯◯  LOW | Important |
| ***AHI (episodes/h) - Infants and small children*** | | | | | | | | | | | | |
| 9 | observational comparisons | not serious ^2^ | serious ^7^ | not serious ^5^ | not serious ^8^ | none | 190 | 190 | - | MD **0.7 more**  (0.6 fewer to 2.1 more) | ⨁◯◯◯  VERY LOW | Important |

MD: mean difference; CI: confidence interval; AHI: apnea/hypopnea index; SpO_2_: percutaneous oxygen saturation

1. Three more studies were not included: Not sufficient data for analysis given
2. Studies in which patients were their own controls.
3. In a number of the studies there was unclear bias regarding representativity, but internal validity was intact, so we did not downgrade for this.
4. Unexplained heterogeneity regarding the size of effect, but a clear effect estimate in favor of the intervention. We upgraded for large effect.
5. Indirectness in population
6. Small cumulative sample size, but clear benefit
7. Unexplained heterogeneity regarding direction of effect, I^2^=90%, we downgraded for this uncertainty
8. CI 95% includes both benefit and harm, but clinically insignificant difference
